# Pharmacological and Toxicological Effects of Phytocannabinoids and Recreational Synthetic Cannabinoids: Increasing Risk of Public Health

**DOI:** 10.3390/ph14100965

**Published:** 2021-09-24

**Authors:** Vidyasagar Naik Bukke, Moola Archana, Rosanna Villani, Gaetano Serviddio, Tommaso Cassano

**Affiliations:** Department of Medical and Surgical Sciences, University of Foggia, 71122 Foggia, Italy; vidyasagar.bukke@unifg.it (V.N.B.); archana.moola@unifg.it (M.A.); rosanna.villani@unifg.it (R.V.); gaetano.serviddio@unifg.it (G.S.)

**Keywords:** synthetic cannabinoids, phytocannabinoids, *Cannabis sativa*, schedule 1 drugs, spice, K2, electronic cigarettes, herbal incense, bath salts, cloud 9, Mojo, John William Huffman (JWH), Hebrew University (HU), MDMB-4en-PINACA, 4F-MDMB-BICA

## Abstract

Synthetic Cannabinoids (CBs) are a novel class of psychoactive substances that have rapidly evolved around the world with the addition of diverse structural modifications to existing molecules which produce new structural analogues that can be associated with serious adverse health effects. Synthetic CBs represent the largest class of drugs detected by the European Monitoring Centre for Drugs and Drug Addiction (EMCDDA) with a total of 207 substances identified from 2008 to October 2020, and 9 compounds being reported for the first time. Synthetic CBs are sprayed on natural harmless herbs with an aim to mimic the euphoric effect of Cannabis. They are sold under different brand names including Black mamba, spice, K2, Bombay Blue, etc. As these synthetic CBs act as full agonists at the CB receptors, they are much more potent than natural Cannabis and have been increasingly associated with acute to chronic intoxications and death. Due to their potential toxicity and abuse, the US government has listed some synthetic CBs under schedule 1 classification. The present review aims to provide a focused overview of the literature concerning the development of synthetic CBs, their abuse, and potential toxicological effects including renal toxicity, respiratory depression, hyperemesis syndrome, cardiovascular effects, and a range of effects on brain function.

## 1. Introduction

The medicinal properties of the *Cannabis sativa* plant were included for the first time in the textbook of pharmacology *Materia Medica* by the Greek and Roman physicians in the first century AD. However, a more accurate description of the physiological effects of this plant was given by the ancient Indian and Chinese writers [1]. *Cannabis sativa* was originally cultivated in central Asia but subsequently spread to most areas of the world. It grows naturally but, due to its pleasurable psychological effects, is frequently cultivated indoors under artificial light [1]. Delta-9-tetrahydrocannabinol (THC) is its most recognised phytocannabinoid and major psychoactive compound [2]. Cannabidiol (CBD) and Cannabinol (CBN) are the other main ingredients and 85 other CBs with different pharmacological effects have been identified and isolated from *Cannabis sativa* [3,4,5].

Following the discovery of the structure and stereochemistry of THC, synthetic CBs were synthesized in the early 1960s to study the pharmacology of CB receptors and to investigate their therapeutic effects [6]. In Europe and the USA, synthetic CBs started to appear in the 2000s and 2008, respectively [7]. In July 2012, synthetic CBs have been classified as Schedule I based on their chemical structures by the Drug Enforcement Administration, which passed the Drug Abuse Prevention Act to decrease the sales and usage of the synthetic CBs [8]. To circumvent this restriction, new synthetic CB compounds were developed, some of which had an inadvertent range of adverse and potentially lethal effects [9]. In the last 20 years, the USA has experimented with legalised access to *Cannabis* and *Cannabis*-derived products for medical and recreational purposes, which has increased their availability in the market and led to increased heavy usage in the adult population [10]. The expanded legal access of *Cannabis*-derived products like chocolate squares, gummy bears, and candies are attracting younger age profile and has increased the incidence of adverse reactions in children [11]. Therefore, synthetic CBs spread easily due to their easy access and intelligent marketing, but they often display variable potency and unpredictable effects. Moreover, their consumption is often undetectable with available standard drug screening tests [12]. Synthetic CBs are manufactured in unregulated laboratories, purchased by local distributors who sell them to the customers by diluting in a solvent [13]. Some synthetic CBs are more toxic than THC with numerous adverse effects, including hypertension, tachycardia, hallucination, emesis, agitation, and seizures [14,15]. *Cannabis* is the world’s most abused drug, and its illicit usage is growing in schools. In the USA, nearly 2.6 million new users under 19 years of age are exposed to *Cannabis* annually [16]. In 2016, the United Nations Office on Drugs and Crime (UNODC) estimated that around 28 million adults (aged 15 to 64) have used *Cannabis* [17].

The World Health Organization (WHO) reported that *Cannabis* use has deleterious effects on the cardiovascular system and progressively it has been registered an increase of THC content in the *Cannabis* from 2–3% to 20% [18]. Since 1998, *Cannabis* has been legalised in 29 states of the USA for medical purposes. In 2017, as per estimations of the *Cannabis* Industry Annual Report, there is a legal market of US$7.97 billion, which is projected to increase by $24 billion by 2025 [19]. There are growing numbers of psychoactive synthetic CBs, which are 10 to 200 times more potent than THC. They are sprayed on harmless herbs, and they are distributed with different market names including Black mamba, K2, spice, Bombay Blue, and fake weed [20]. This explosive increase in the availability of CB variants is, unfortunately, increasing the incidence of deaths and fatalities [21].

In the context of the increasing popularity of synthetic CBs around the world, there is a rise in toxicity cases due to the usage of recreational *Cannabis* and its synthetic analogues. The main aim of this review is to discuss the adverse effects of synthetic CBs including kidney injury, hyperemesis syndrome, cardiovascular effects, respiratory depression, and brain damage.

## 2. Methodology

**Figure 1:**
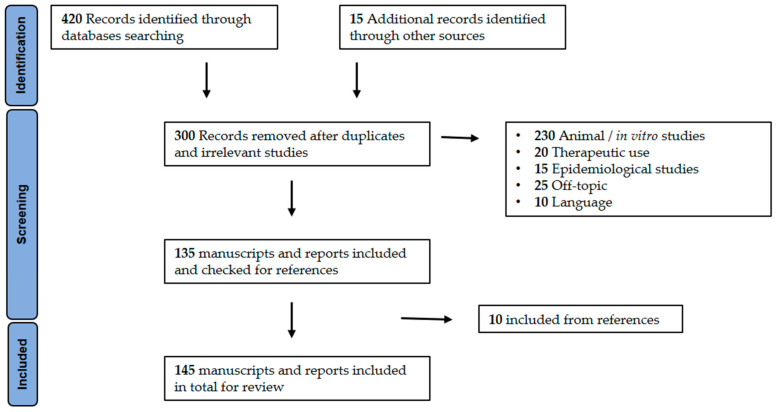
Flow diagram of study identification, screening, and selection.

A literature search was conducted on multidisciplinary research databases such as PubMed, Scopus, and Web of Science and international agencies or institutional websites including the WHO, UNODC, Centers for Disease Control (CDC), U.S. Department of Justice, US FDA, US Drug Enforcement Administration (DEA), US National Institute of Health (NIH), EMCDDA, and European Medicines Agency (EMA) to identify the most relevant literature. The search terms used alone or in combination were: “synthetic cannabinoids”, “*Cannabis sativa*”, “Schedule 1 drugs”, “phytocannabinoids”, “spice”, “K2” “cannabinoids”, “electronic cigarettes”, “adverse effects”, “cloud 9”, “Bath salts”, “mojo”, “John William Huffman (JWH)”, “Hebrew University (HU)”, “EMCDDA”, “MDMB-4en-PINACA”, “4F-MDMB-BICA”, “toxic effects” and “herbal incense”. Only the articles written in English were selected. All the articles were reviewed independently by the authors to determine their relevance in the framework of the current study. Details of the literature search are listed in Figure 1.

## 3. Endogenous Cannabinoids and Receptors

**Figure 2:**
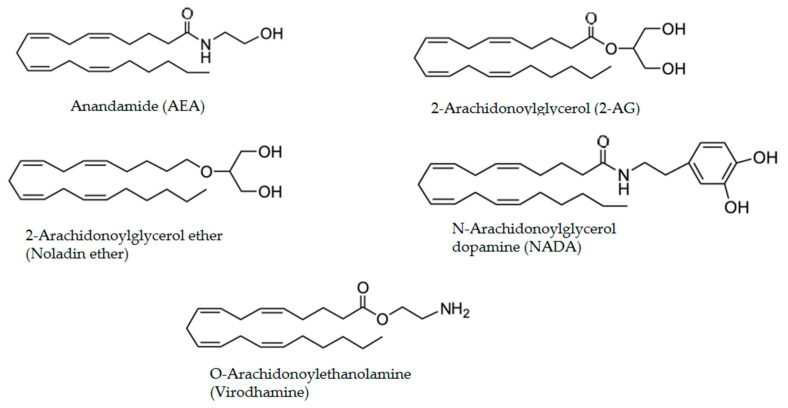
Chemical structures of the endocannabinoids.

*N*-arachidonoyl-ethanolamine (AEA) and 2-arachidonoyl glycerol (2-AG) are the two most studied endocannabinoids (eCBs), although other eCBs also exist, including 2-arachidonyl glycerol ether (Noladin ether), O-arachidonoyl ethanolamine (virodhamine), N-arachidonoyl glycine, and N-arachidonoyl dopamine (NADA) [22,23,24,25] (Figure 2).

AEA and 2-AG are lipophilic molecules and are synthesized de novo by cleavage of their membrane lipid precursors *N*-arachidonoyl-phosphatidylethanolamine (*N*-ArPE) and *sn*-1-acyl-2-arachidonoylglycerols (DAGs) respectively [26]. The enzymes *N*-acyl-phosphatidylethanolamine-selective phospholipase D (NAPE-PLD) and diacylglycerol lipase (DAGL) biosynthesise AEA and 2-AG, respectively; the enzymes fatty acid amide hydrolase (FAAH) and the monoacylglycerol lipases (MAGL) are involved in the degradation of AEA and 2-AG, respectively [27]. These enzymes, together with CB receptors and eCBs, are key components in the eCB system. AEA, named anadamide after the Sanskrit word ananda or “bliss”, was discovered in 1992 and functions as a partial agonist at CB type 1 (CB1) receptors but is inactive at CB type 2 (CB2) receptors [22]. AEA also interacts with other targets including the GPCR112, GPCR55, transient receptor potential vanilloid-1 (TRPV1) channels, alpha7-nicotinic acetylcholine receptors, T-type Ca^2+^ channels, Na^2+^ channels and voltage-gated K^+^ channels [28,29].

2-AG was identified in 1995 and acts as a full agonist at both CB1 and CB2 receptors [26,27]. The CB1 receptor was discovered on the basis of its responsiveness to THC and was first cloned in 1990 [22,30]. It is widely distributed in the body and the brain, CB1 is expressed at higher densities in basal ganglia, hippocampus, cortex, and cerebellum [31] the motor function, cognition, memory, and analgesia [32]. Significant levels of CB1 receptors are expressed in peripheral tissues including the adrenal gland, heart, lungs, prostate, bone marrow, thymus, tonsils, ovary, and testes [33]. The CB2 receptors were isolated in differentiated myeloid cells and cloned in 1993 [34]. CB2 receptor shares 44% amino acid homology with CB1 [35], is mainly expressed in macrophages, spleen, tonsils, thymus, leukocytes, lungs, and testes, and its stimulation is attributable to the anti-inflammatory and immunomodulatory actions of CB [36]. Both CB receptors are members of the G protein-coupled receptor (GPCR) family and are coupled to pertussis toxin (PTX)-sensitive G_i/o_ protein, suppress adenylyl cyclase (AC) and the formation of cyclic adenosine monophosphate levels (cAMP) upon receptor activation. CB1 receptor was found in both glutaminergic and GABAergic terminals and its activation at presynaptic levels can suppress the excitatory and inhibitory neurotransmission, respectively [37,38,39,40]. Synthetic CB receptor agonist is linked to the mitogen-activated protein kinase (MAPK) pathway, which results in phosphorylation of nuclear transcription factors influencing cellular transcription, translation, motility, shape, proliferation, and differentiation. Prolonged phosphorylation of CB1 receptors may lead to desensitisation and internalization that could impact respiratory depression indirectly [41,42].

Like the CB1, the CB2 receptors inhibit the activity of AC through their Gi/Go_α_ subunits. Through their G_βγ_ subunits, CB2 receptors are also known to be coupled to the protein kinase C (PKC) leading to the activation of the MAPK and extracellular signal-regulated kinase (ERK) phosphorylation [43]. Therefore, CB2 receptors through the MAPK-ERK pathway can modulate a complex and highly conserved signal transduction pathway, which regulates several cellular processes in mature and developing tissues [43]. Moreover, CB2 receptor activation induces intracellular Ca^2+^ release from the endoplasmic reticulum, as well as an increase in mitochondrial Ca^2+^, via the phospholipase C (PLC) activation and inositol 1,4,5-triphosphate (IP_3_) production [44].

## 4. *Cannabis sativa*

*Cannabis sativa*, an important plant containing more than 600 pharmacologically active constituents, has been used medicinally for several hundred years and has a range of other applications due to its cellulose and woody properties. *Cannabis* is rich in phytocannabinoids, which are C21 or C22 groups of terpenophenolic compounds predominantly rich in THC, Δ9-Tetrahydrocannabivarin (THCV), CBD, CBN, cannabigerol (CBG), cannabichromene (CBC), and Cannabidivarin (CBV) [45]. The chemical structures of these above-written compounds and their properties are included in Table 1. Herbal preparations of cannabis for medical use are commercially available in the form of vaporizers, buccal sprays, oral capsules, decoctions, and oils [46].

THC is the major cannabinoid in *Cannabis sativa* and is most significant due to its pharmacological and toxicological properties. This plant is also rich in phenolic compounds such as flavonoids belonging to flavone and flavanol subclasses [47]. *Cannabis* contains about 120 phytocannabinoids and until recently eleven chemical classes of phytocannabinoids have been identified. Among them, THC class is the largest portion comprising 17.3% of the total phytocannabinoid content [48]. Phytocannabinoids can be classified into tricyclic (THC, THCV, and CBN), bicyclic (CBD, CBC, and CBV), and others (CBG).

## 5. Therapeutic Effects of Phytocannabinoids and Synthetic Cannabinoids

Despite the addictive potential of marijuana, *Cannabis sativa* has a very long history of medicinal usage. THC was isolated from hashish in 1964 by Raphel Mechoulam at Hebrew University, which led to the discovery of CB receptors. The functions of eCB, and the use of CB1 and CB2 receptors as targets for medical and other recreational purposes [76]. Synthetic CB agonists are increasingly used for the treatment of human pathological conditions and several CB antagonists are under clinical evaluation. Marijuana contains THC at different concentrations in the range of 40 to 80% and appears like honey or butter (brown or gold colour) and is usually consumed orally in the form of food or drink products, smoking by use of oil or water pipes. E-cigarettes/vaporizers are often preferred due to their smokelessness, odourless, and ability to hide from consuming marijuana concentrate [77]. Besides, the marijuana plant contains 60 cannabinoid-like structures out of its total 600 chemicals [78].

In both the brain and periphery, the eCB system modulates many physiological processes, which have some beneficial therapeutic effects in the treatment of pain, inflammation, epilepsy, cancer, eating disorders, and spasticity. CBs are effective in treating postoperative pain, cancer, and spinal cord injury [79]. Dronabinol, which is an appetite-increasing drug used in HIV patients, is a commercially available form of THC [80].

Some evidence also showed that in animal models, *Cannabis* smoke alleviated the tremors and spasticity associated with MS [81], and some clinical studies have shown that CBs could be used in combination therapy for treating cancer [82]. CBs exert inhibitory effects on glutamate, reactive oxygen species (ROS), and tumour necrosis factor indicating that they play a crucial role as neuroprotective agents [83,84,85,86]. Moreover, CBs may offer a potential treatment for Parkinson’s disease (PD) as they inhibit the excitatory effects of glutamate [87,88,89,90].

It has been demonstrated that CBs exert anti-cancer properties in a range of cancers [91]. CBs were preclinically determined to be potential antineoplastic agents against pancreatic cancers [92], breast cancers [93], gastric cancers [94], prostate cancers [95], leukaemia [96], skin carcinomas [97], cervical cancers [98], colon cancers [99], non-small cell lung cancers [100], hepatocarcinomas [101], bladder carcinomas [102] and multiple myeloma [103]. CBs, as anticancer treatments, can induce apoptosis [104], autophagy [105], and antiproliferative effects [106].

THC with its synthetic analogues nabilone (Cesamet^®^; Valeant Pharmaceuticals, Irvine, CA, USA) and dronabinol (Marinol^®^; Solvay Pharmaceuticals, Marietta, GA, USA) have been used to suppress nausea and vomiting associated with chemotherapy [107]. In this context, THC is thought to exert its pharmacological effects by decreasing 5-HT3 receptor activity [108]. In animal models, THC has demonstrated considerable neuroprotective effects and is capable of ameliorating the symptoms of neurodegenerative diseases, including PD, AD, HD, MS, and Amyotrophic Lateral Sclerosis (ALS) [108,109,110,111,112,113,114]. It has also been demonstrated that THC and cyclooxygenase-2 (COX-2) inhibitors can reduce Aβ plaques in degenerated neurons of AD animal model [115]. Following THC treatment, some mouse models of MS showed improved spasticity and tremors [116]. In clinical trials of MS patients, THC exerted both decreased urinary incontinence and antispasticity effects [117]. Although THC has its therapeutical effects, it also has psychoactive properties; alternately, CBD is less toxic than THC to humans and has been recognised as a nonpsychoactive compound [115].

The psychoactive properties of THC suggest that it may have considerable potential in the treatment of a range of psychological/psychiatric conditions; CBD exerts a wide range of positive therapeutic effects in psychosis, anxiety, and depression-like behaviours by neuroprotection and inhibiting neuroinflammatory responses. CBD can also act independent of cannabinoid receptors by modulating antioxidant mechanisms in PD animal models and attenuates dystonia [88,89,90]. In MS, CBD treatment can exert a neuroprotective role by diminishing inflammation through adenosine A2 receptors [115]. CBD reduces neuroinflammation and axonal damage of oligodendrocyte progenitor cells (OPC), which increases differentiation into new myelinating oligodendrocytes. Synthetic CBs protect the OPCs by controlling the stress response of the endoplasmic reticulum which modulates inflammatory stimuli [118]. Sativex^®^, an oromucosal spray (GW Pharmaceuticals, Cambridge, UK), showed positive results in clinical trials for MS and it is marketed in 16 countries outside of the USA. It has antispasmodic and analgesic properties containing a 1:1 ratio of plant extracted THC and CBD [119]. In 2007, a meta-analysis study conducted in MS treatment using Sativex^®^, CBD, and Marinol^®^ showed that Sativex^®^ has the highest efficacy in reducing the neuropathic pain with dizziness as the adverse effect [120]. USFDA approved synthetic drugs such as Marinol^®^ (synthetic THC), is used to treat anorexia associated with weight loss in patients with AIDS (Acquired Immunodeficiency Syndrome), Cesamet^®^ (nabilone), and Syndros^®^ (synthetic THC) used for the treatment of nausea and vomiting due to chemotherapy [121].

## 6. Synthetic Cannabinoids as Drugs of Abuse

*Cannabis* has been used as a drug for centuries but in the nineteenth century, CBN was isolated, and its structure was partially described in 1932 [122]. In 1964, Raphael Mechoulam characterised the structure of THC, which lead to the development of THC analogues with small modifications in their structure which produce new moieties with similar activity, resulting in the production of synthetic CBs as potential drugs of abuse [123]. Since 2004 synthetic CBs have been sold in European countries such as Germany, Austria, Switzerland over the internet, which enabled very easy access to synthetic CBs without any age restriction [124].

Synthetic CBs are a novel range of psychoactive substances, which have similar effects like THC, and their potency depends on their structure and volume of consumption. It is believed that synthetic CBs are 800 times more potent than *Cannabis* [125]. The most commonly used name for synthetic CBs is spice, which is marketed as a relaxing herbal blend hiding its true composition. It is sold in black markets at a higher price than marijuana and gained its popularity due to its legality and negative drug test results. In mid-2008, spice’s popularity reached peak levels in Germany, leading to an epidemic of accidents and mental disorders in spice users [126]. Spice is known as K2 in the USA [127], which rapidly became a drug of abuse and as a consequence got banned in 2010 [128].

Initial attempts failed to explain the narcotic effects of spice products because the manufacturers claimed that it has a herbal composition. However, it was gradually understood that the herbal ingredients do not induce significant psychoactive effects [129]. Suspicion arose that spice products had been mixed with synthetic CBs, but there was insufficient analytical data to reach this conclusion. These blends contain the JWH series of drugs developed by John W. Huffman while working at Clemson University and the HU series of drugs developed at HU [130]. In 2008, the German pharmaceutical company THC Pharm has isolated a synthetic CB JWH-018 from 3 spice varieties of the synthetic CBs [13], and two other research teams at Freiburg University (Germany) [124]. The National Scientific Institute of Health (Japan) [131] identified the presence of C8 homolog of synthetic CBs including JWH-018, CP 47497 which were subsequently made illegal by the German regulatory bodies and other European countries [129]. To stay ahead of the enforcement measures, illegal drug teams synthesized new kinds of synthetic CBs which were similar to the prohibited and banned chemical structures including JWH-073, which is a butyl homolog of JWH-018. New synthetic CBs are structurally related to the prohibited molecules with slight changes. To overcome this issue, the Russian Government released Resolutions of No.882 and No.1178 defining the term “drug derivative”. Nevertheless, the drug traffickers then synthesized new molecules with a range of innovative structural modifications [132].

In contemporary society, large-scale synthetic CBs were manufactured illegally for recreational purposes due to their high potency and undetectability by conventional drug screening tests. Alterations and substitutions of the chemical moieties in THC produced more than 700 synthetic CBs and some of them have active metabolites [133]. JWH-018 is a synthetic CB, which is a full agonist to CB1 and CB2 receptors and can cause hallucinations, vertigo, paresthesias, shaking, shivering, hypertension, dry mouth, vomiting, mydriasis, hypokalemia, extrasystoles, tachycardia and conjunctival hyperemia [134]. Recent data shows that K2, the synthetic CB, can cause ischemic stroke [135] and further data is needed to understand the toxic effects of synthetic THC on brain mitochondria [136]. Liquid chromatography-tandem mass spectroscopy (LC-MS) helps in analysing the synthetic CBs in serum samples derived from people suspected to use spice. The spice varies in its chemical constituents as per the supplier and it can include toxic chemicals like oleamide, linoleic acid, palmitic acid, palmitoylethanolamide, eugenol, thymol, acetyl vanillin, benzyl benzoate, α-tocopherol, vitamin E, and β_2_-adrenergic agonist [137]. JWH-018 and CP 47497 were the first CBs to be identified in spice using Gas chromatography-mass spectrometry (GC-MS) chromatography [138]. It has been demonstrated in mice that co-administration of JWH-018 and JWH-073 can produce addictive, synergistic, and antagonistic interactions [139].

Synthetic CBs were sold as a legal replacement for *Cannabis* and attracted a lot of attention for having the same powerful intoxicating effects. Moreover, there was also an increase in availability of e-liquid products containing a mixture of cannabinoids in a solvent that is vaped using an electronic cigarette. Smuggling of cannabinoids into prison in some countries is facilitated by impregnating paper (such as letters, greeting cards, photographs, children’s drawings) with cannabinoids and then vaped using electronic cigarettes, this can pose a high risk of poisoning due to high degrees of cannabinoid variability in different parts of the paper [140]. Many adverse health issues have been reported in USA, Russia, Canada, and Europe due to the misspelling or adulteration of the product. For instance, synthetic CBs in Europe are sold as ecstasy, while in the USA and Canada, opioids like heroine are adulterated with synthetic CBs [141].

As previously mentioned, the limited availability of selective and sensitive rapid analytical methods for screening the synthetic CBs is representing a challenge in elucidating the heterogenous structures. Colorimetric and immunochemical assays were found to be inappropriate for rapid and specific detection due to frequent cross-reactivity and can take more time for the production of antibodies. While chromatographic assays proved to be more suitable due to high flexibility, sensitivity, and selectivity even at low doses. The hyphenated techniques like GC-MS and/or LC-MS are the best promising tools but are expensive [142].

In New Zealand and Australia, a potent cannabinoid known as Kronic came into the market after spice and Dream in 2011, and unfortunately caused the death of a huge number of young people [143]. New synthetic CBs, such as JWH-250, JWH-398 [129,144], AM-694 [145], RCS-4 [146], AM2233 [147] are constantly emerging in the market with new chemical moieties to prevent their identification and regulation as illicit substances [132]. Due to their potential toxicity and abuse, the USA government has listed some synthetic CBs under schedule 1, which contain drugs, substances, or chemicals defined by the federal government with no currently accepted medical use and high potential of abuse. Some schedule 1 synthetic cannabimimetic agents are listed in Table 2 with their structures and other relative information [148].

According to the recent report released in December 2020 by EMCDDA, 207 emergent synthetic CBs were monitored in the drug market since 2008 which include nine compounds reported for the first time up until the end of October 2020. Besides, from 2011 to 2015, 27 compounds appeared on average every year in Europe but, from 2016 this number dropped to around 10 [160]. Seizures related to synthetic CBs reported by the European Union (EU) Early Warning System were 19,705 in 2019 representing 46% of the total seizures during the year and in which, synthetic CBs were mainly detected in herbal plant material (5977 cases-138 kgs) in the form of powder (728 cases-84 kgs). However, in recent years, a sharp decrease has been observed in seizing the powder quantity and herbal materials containing synthetic CBs in the EU. But two synthetic CBs MDMB-4en-PINACA [161] and 4F-MDMB-BICA (or 4F-MDMB-BUTICA) [162] were observed during 2020, increasing the potential threat to public health in EU and their details are reported in Table 3.

The aim of developing the synthetic CBs was for research purposes but the published scientific literature on the synthesis of synthetic CBs has been misused by criminally inclined chemists to produce huge amounts of synthetic CB products for illicit commercial use [164] and was sold under the brand names Spice, K2, Black Mamba, Scooby snax [165], herbal incense, Cloud 9, and Mojo [6].

**Figure 3:**
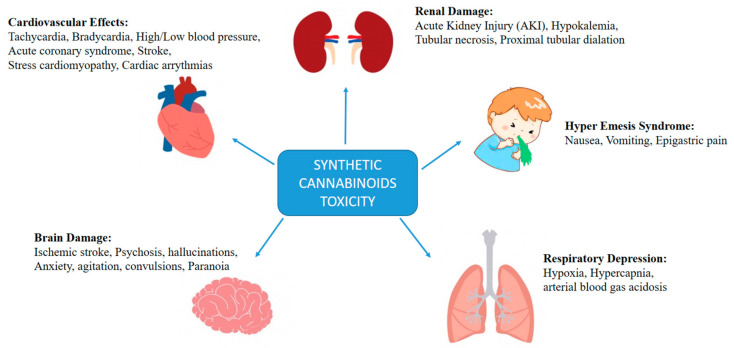
Toxicity of synthetic cannabinoids and their adverse effects.

Synthetic CB intoxication leads to different physiological and psychological effects including psychosis [166], respiratory depression [42], renal toxicity [167], hyperemesis syndrome [168], cardiovascular effects [169], gastrointestinal problems [170], seizures [171], and acute cerebral ischemia [171]. Therefore, synthetic CB usage is associated with toxic effects on the body and multiple organ failure. Figure 3 describes the adverse effects of synthetic CBs, which are further discussed in this review.

## 7. Toxicological Effects of Synthetic Cannabinoids

### 7.1. Renal Injury

Kidneys are the vital organs in maintaining the fluid homeostasis of the body, regulating and filtering the minerals from the blood, producing hormones, that are important in producing the red blood cells (RBC), promoting bone health, and regulating blood pressure. Kidney damage is associated with a higher rate of morbidity and mortality due to its involvement in significant functions of the body. Synthetic CBs, which are often used as recreational drugs, are emerging in the market due to their greater availability at cheaper prices with comparable psychoactive effects as *Cannabis* with adverse renal effects [172]. Renal biopsy studies revealed that people addicted to synthetic CBs are suffering from tubular necrosis and proximal tubular dilatation [167]. Long-term use of synthetic CBs damages the kidney with abnormal urinalysis and increased levels of creatinine levels in blood [157].

Evidence suggests that both CB1 and CB2 receptors are involved in the pathogenesis of acute kidney injury (AKI) in mice. CB1 expression has been found in different regions of the nephron in rodents, including afferent arterioles, efferent arterioles [173], glomerulus [174], thick ascending loop of Henle [175], tubular epithelial cells [176], and cultured mesangial cells [177]. CB1 receptors have been detected in proximal convoluted tubules, distal tubules, and intercalated cells of the collecting duct in human kidneys [178]. CB2 receptors are expressed in podocytes [179], proximal tubule cells [180], mesangial cells of human and rat renal cortex samples [181].

The eCB system in kidneys plays an important role through its increased CB1 receptor activity contributing to hemodynamic abnormalities and dysfunction. A series of studies in nephrotoxic models of cisplatin-induced renal injury demonstrated that there are detrimental effects due to CB1 activation and protective effects by CB2 activation. CB1 activation results in increased expression of oxidative/nitrosative stress marker, which activates pathways such as MAPK, P38, c-Jun N-terminal kinase pathways leading to apoptotic cell death and inflammation [182]. On the contrary, CB2 activation leads to reduced proapoptotic signalling, involved in anti-inflammatory effects by decreasing the release of cytokines [183].

Buser and colleagues reported two clinical cases regarding the effect of synthetic CBs on kidney function. In one of the cases, a 17-year-old patient smoked a synthetic CB called Clown Loyal and presented with symptoms of flank pain, emesis, and oliguria. Later, the patient was treated with furosemide for oliguria and intravenous methylprednisolone (500 mg for 3 days) for suspected acute interstitial nephritis, followed by oral prednisone (30 mg twice daily). Renal biopsy conducted on the third day exposed the real condition of the kidney that the patient is having an acute tubular injury with mild interstitial nephritis. In another case, 15-year-old reported to have discomfort and renal ultrasound revealed bilateral hyperechoic kidneys with poor corticomedullary differentiation [184]. Illicit use of synthetic CBs increased the number of illness cases. In addition, 16 cases were reported of AKI in six states of the USA in March 2012 [185], and four cases of oliguric AKI in 2013 [167]. Acute tubular necrosis seems to be the aetiology of AKI in patients [186].

### 7.2. Cannabinoid Hyperemesis Syndrome

Cannabinoid hyperemesis syndrome (CHS) is a condition with cyclic vomiting due to high doses of *Cannabis*. *Cannabis* has been used for many years, but CHS was first reported in 2004 [187]. The patients suffering from this condition had to undergo a lot of expensive medical tests for symptom management. The pathophysiology of CHS is less known, which hinders proper treatment of this condition [188]. CHS has been given less importance and underestimated but now, many cases have been identified in recent years showing the widespread use of *Cannabis* throughout the world. There are some sceptical and unclear circumstances under which *Cannabis* suppresses emesis in some people, and induces in others. This needs to be clarified. It is difficult to understand the exact pathophysiology of CHS, as some data suggest that it is due to dysregulation of the eCB receptors CB1 and CB2 in the brain and gastric system [189], while other data suggest that interaction of CBs with CB1 in gastrointestinal tract changes the gastric motility. Widespread use of synthetic CBs increases CHS cases day by day as they are potent against CB1 receptors [190].

A case report of a 37-year-old African American male who is suffering from intermittent symptoms like nausea, vomiting and epigastric pain for the last 14 years has a history of long-term use of *Cannabis* and to relieve the symptoms the patient applies heat to the abdomen [191]. It remains a puzzle how CBs induce hyperemesis despite its antiemetic property demonstrated in clinical and animal models, by stimulating CB1 receptors [192]. Hundreds of metabolites formed from the active components of *Cannabis* and the non-active components could trigger the cyclic hyperemesis following chronic abuse [193]. Abdominal heat application with hot showers helps in the redistribution of heat around the gastrointestinal tract and relieves the symptoms of CHS [194]. One case report suggests that chronic use of CBs may turn CHS into Cyclic Vomiting Syndrome (CVS) [191]. A study conducted by Simonetto and colleagues on 98 patients concluded that CHS symptoms occur in patients who use *Cannabis* frequently for many years and start to decline after the cessation of the *Cannabis* use [195].

Nausea and vomiting associated with cancer chemotherapy can be treated with THC (Dronabinol) and the synthetic cannabinoid nabilone which were approved for medical use in the USA. The difference between anti-emetic doses and adverse (psychic) effects of these drugs is too narrow, which limits its usage as anti-emetic in a clinical context. However, the introduction of 5-HT_3_ receptor antagonists as new anti-emetics emerged as a better option to replace cannabinoids [196].

### 7.3. Cardiovascular Effects

Smoking *Cannabis* causes an immediate increase in heart rate lasting more than an hour, and in half an hour there is an increase in serum norepinephrine levels [197]. Increased supine systolic blood pressure is noticed on acute exposure to *Cannabis* [198] that may induce atrial fibrillation [199]. *Cannabis* smokers have reduced levels of oxygen in their blood due to increased levels of carboxyhemoglobin [200]. Moreover, *Cannabis* is found to be a source of ROS, which increases cellular oxidative stress [201].

Cardiovascular cases increased from 1.1% in 2006 to 3.6% in 2010. The commonly reported cardiovascular cases of *Cannabis* users are acute coronary syndrome (ACS), stroke, stress cardiomyopathy, cardiac arrhythmias, and death [202]. Nearly a five-fold increase in the risk of acute myocardial infarction (AMI) after *Cannabis* exposure was described [203]. CBs can reduce myocardial contractility by acting on CB1 receptors [204] and many cases have been reported regarding the rhythm abnormalities in *Cannabis* users [205]. It has been reported that young users (14 years old age) were found to have atrial fibrillation after *Cannabis* smoke [206].

Acute myocardial infarction in marijuana smokers was increased, 4.8-fold higher during the first hour of exposure, and declined rapidly [203]. An investigation revealed that recreational marijuana usage by young adults (age 25–34 years) led to a 17% increased hospitalisation due to ischaemic stroke and 18% due to aneurysmal subarachnoid haemorrhage [207]. Other cases presented with acute myocardial infarction after smoking spice or k2 with chest pain in 16-year-old boys [169].

### 7.4. Respiratory Depression

Synthetic CBs act on peripheral receptors, such as chemoreceptors and baroreceptors, increasing the resistance on bronchial air passage. Activation of CB1 receptors could be a possible reason for respiratory depression by stimulating signalling pathways linked to G-protein coupled receptor, MAPK which leads to the suppression of excitatory and inhibitory neuronal activity [41].

The effect of synthetic CBs in respiration has not been studied extensively on humans and research on rats had shown that they affect the respiratory rate and can cause hypoxia, hypercapnia, and arterial blood gas acidosis [208]. Inhalation of synthetic CBs causes the release of harmful chemical gases which damage the bronchial epithelial lining and damages the protective surfactant layer in alveoli leading to changes in the gaseous exchange causing hypoxia and acute respiratory distress [209]. The chronic use of synthetic CBs leads to dependence syndrome, withdrawal symptoms, and psychiatric symptoms [145]. Severe respiratory depression, pneumothorax, and acute respiratory distress syndrome (ARDS) can be developed after synthetic CBs use. In a 25-year-old boy in Turkey, it was found that after consumption of synthetic CBs, the pulmonary parenchymal tissue was damaged, causing vasoconstriction and endothelial dysfunction [210].

Synthetic CBs can act as full agonists, partial agonists, and inverse agonists at CB1 and CB2 receptors, and have different levels of potency, efficacy, affinity, selectivity, and molecular activity [211]. Contrarily, natural THC shows only partial agonistic activity [212].

Marijuana smoke is comparatively more harmful than tobacco smoke to the lungs and induces bronchial hyperresponsiveness, pulmonary inflammation, emphysema, and tissue destruction independent of CB1 activation. A single marijuana cigarette per day is equivalent to one package of tobacco cigarettes in inducing the risk of malignancy tumours every year. Twice the concentrations of harmful chemicals including phenol, naphthalene benzanthracene, acetaldehyde, hydrogen cyanide, and ammonia are present in marijuana compared to tobacco smoke. These toxic irritants activate peptidergic sensory nerves which induces inflammation. In blood carboxyhemoglobin levels are five times higher in marijuana smokers compared to tobacco smokers [213]. A 29-year-old male patient who smoked bonzai, a synthetic CB derivative, for 2 years suffered from pulmonary embolism, a clinical condition that is associated with a higher rate of morbidity and mortality [214]. Similarly, another 32-year-old female patient also suffered pulmonary embolism due to the use of synthetic CBs [215].

### 7.5. Effects on Brain

In the brain, CB1 receptors are extensively distributed in regions like the hippocampus, basal ganglia, cortex, amygdala, and cerebellum. *Cannabis* induces dose-dependent toxicological changes in these brain regions [216]. There are changes in grey or white matter density in different regions of the brain including the frontal and parietal lobes [217] and degenerative changes in the hippocampus and amygdala are reported more often [218]. These findings suggest that *Cannabis* use leads to changes in the morphology and function of brain structures involved in learning and memory [219,220]. Battistella and colleagues showed that there was a decrease in grey matter volume in regions, including the temporal cortex, temporal pole, parahippocampal gyrus, left insula, and orbitofrontal cortex in regular *Cannabis* users as compared with occasional users [221]. The same results have been found in animal studies, which further corroborate that *Cannabis* exposure results in volume reduction of CB1 rich regions [216].

Functional magnetic resonance imaging (FMRI) studies also demonstrate that there are alterations in core regions of the brain including the ventromedial prefrontal cortex (vmPFC), insula, and orbitofrontal cortices, which are linked to motivation and decision making [222]. However, in addition to changes in the polar region of the brain, changes have also been noted in the medial temporal cortex. The hypothesis of the reduction in grey matter volume suggests that it is due to abnormal pruning of the synaptic connections [223], which damages the brain maturation process. The exogenous CBs interfere with the normal functioning process of the eCBs and alter the pruning activity of the synapses in brain regions including the cerebellum [224] and prefrontal cortex [37,38,40,85,225].

In a 50-year-old male, fluid attenuation inversion recovery (FLAIR) imaging studies have shown that synthetic CB usage led to abnormally intense signals in the hippocampus, basal ganglia, bifrontal cortex, cerebral peduncles, posteroinferior cerebellar hemispheres, and cerebellar vermis [226]. Other designer drugs like “Bath salts”, which have similar effects to cocaine, induce acute intraparenchymal and subarachnoid haemorrhage as well as ischemic infarction [227]. Bath salt intoxication in a 36-year-old man led to delayed encephalopathy, dysautonomia, fulminant hepatic failure, and renal failure from severe rhabdomyolysis [228], and in a 14-year-old girl, this was accompanied by hyponatremia [229].

**Figure 4:**
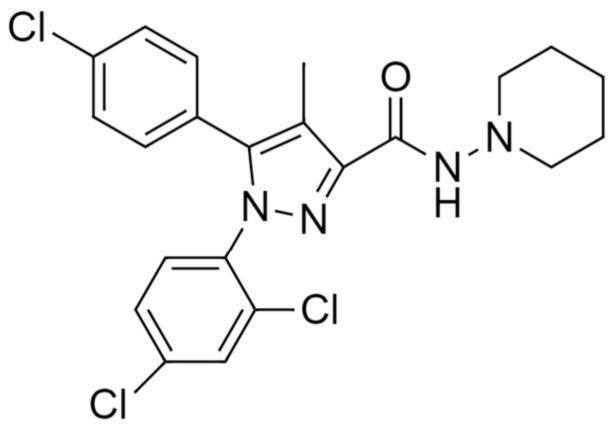
Rimonabant (SR141716A)-Chemical structure of CB1 selective antagonist drug.

Synthetic drugs that selectively act as antagonists of CB1 or CB2 receptors [230], and rimonabant (SR141716A), a selective CB1 receptor blocker [231], has been used widely in CNS (Figure 4). In mice, it has been observed that direct microinjection of synthetic CB CP55940 into the cerebellum did not impair motor coordination [232]. Administration of THC, synthetic CBs, and anandamide in animals caused deficits in short-term memory in a spatial learning task [37,220,233].

To evaluate the effect of synthetic THC, research was performed on isolated rat brain mitochondria, and it induced negative effects on mitochondrial respiratory chain complexes I, II, III and increased the leakage of free radicals on exposure to THC. Moreover, mitochondria also participate in the overproduction of ROS in the presence of THC. Thus, the brain is vulnerable to oxidative stress and mitochondrial dysfunction, which increases neuronal damage and leads to the chance of stroke [140]. In other research, it has been reported that HU210, a synthetic analogue of THC, induces a spatial deficit in the Morris water maze test in learning reference memory [234].

## 8. Conclusions

Phytocannabinoids from the herb *Cannabis sativa* have been used for thousands of years for both medicinal and recreational purposes. Characterisation of the endocannabinoid system in the past two decades has developed an enhanced understanding concerning the roles of CB1 and CB2 receptors, which made it possible to use plant-derived cannabinoids in therapeutic implications. USFDA approved synthetic drugs such as Cesamet^®^ used in severe nausea and vomiting, Marinol^®^ and Syndros^®^ used in AIDS-related anorexia, and antiemetic. Sativex^®^, an oromucosal spray used for the treatment of MS is marketed in 16 countries outside of the USA.

Elucidating the structure of THC in 1964 led to the development of THC analogues with similar activity and increased the production of synthetic CBs. Though the aim of developing the synthetic CBs was for research purposes, it was misused by clandestine chemists for criminal activities and currently, they are manufactured illegally on a large scale for illegal commercial use. Synthetic CBs have evolved and become popular rapidly in the world of recreational drugs of abuse due to their psychoactive properties. In recent times, many SCs have been banned and placed under the schedule 1 category in the USA because of their potentially harmful effects. However, new synthetic CBs with modifications in their chemical structure are available in the market with increased potential toxicity that threatens public health. Adverse effects associated with synthetic CBs include seizures, cardiovascular effects, kidney injury, respiratory depression, hyperemesis syndrome, gastrointestinal problems, cerebral ischemia, and multiple organ failure leading to death. Therefore, there is an urgent need to stop the spread and usage of these synthetic CBs in various forms and to further understand the toxic effects of synthetic CBs to develop treatments for intoxication.

## Figures and Tables

**Table 1:** List of phytocannabinoids with their structures, *K_i_,* and key findings.

Name	Structure	*K_i_/*µM	Key Findings
Δ9-trans-Tetrahydrocannabinol	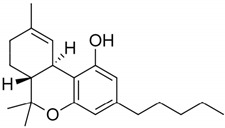	CB1—0.005 ^a^ 0.008 ^b^ 0.013 ^c^ CB2—0.003 ^a^ 0.0017 ^b^ 0.0068 ^c^ [45]	THC inhibits Alzheimer’s disease (AD) pathology (by competitively inhibiting acetylcholinesterase enzyme and beta-amyloid (Aβ) peptide aggregation) [49] As analgesic for neuropathic pain [50,51] Bronchodialator effect on asthma patients [52] Effective to treat intractable cholestatic pruritus [53] Potent against methicillin-resistant *Staphylococcus aureus* (MRSA) strains (MIC- 2 µg/mL) * [54] Inhibit the proliferation of a hyper-proliferating human keratinocyte cell line in the treatment of psoriasis [55]
Δ9-Tetrahydrocannabivarin	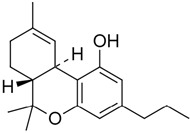	CB1—0.075 ^d^ 0.0047 ^e^ CB2—0.225 ^a^ 0.145 ^a^ [45]	Exerts antiepileptiform and anticonvulsant properties in adult rats [56] Ameliorates insulin sensitivity and can be used to treat obesity-associated glucose intolerance [57] Δ8-Tetrahydrocannabivarin has potent anti-nicotine effects [58] Potent against MRSA strains (MIC—4 µg/mL) * [54] As a potential therapeutic benefit for the management of obesity and diabetes [59]
Cannabinol	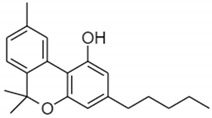	CB1—0.069 ^f^ 0.012 ^g^ CB2—0.016 ^g^ 0.07 ^f^ [45]	Potent against MRSA strains (MIC—2 µg/mL) * [54] Inhibit the proliferation of a hyper-proliferating human keratinocyte cell line in the treatment of psoriasis [55]
Cannabidiol	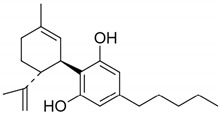	CB1—4.3 ^e^ 1.45 ^g^ CB2—2.86 ^f^ 0.37 ^g^ [45]	Potent against MRSA strains (MIC—2 µg/mL) * [54] Inhibit the proliferation of a hyper-proliferating human keratinocyte cell line in the treatment of psoriasis [55] Potent inhibitor of transporter ABCC1 or MRP1 that helps in accumulation of anticancer drugs in cells [60] Protect against Aβ neurotoxicity in AD [61] Inhibits tau hyperphosphorylation in AD [62] Anti-arthritic by targeting synovial fibroblasts [63,64] Fluorinated derivatives of cannabidiol shows therapeutic activity as anxiolytic, antidepressant, antipsychotic, and anticompulsive [65] Prevents post-ischemic injury via HMGB1-inhibiting mechanism [66]
Cannabidivarin	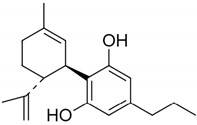	CB1—14.7 ^g^ CB2—0.57 ^g^ [45]	Ameliorates autism-like behaviours, restores endocannabinoid signaling and neuroinflammation [67] Anti-convulsant [68] Potent against MRSA strains (MIC—8 µg/mL) * [54]
Cannabigerol	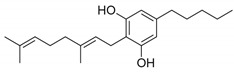	CB1—0.896 ^g^ CB2—0.153 ^g^ [45]	Anti-oxidant properties in macrophages [69] Can be used to treat inflammatory bowel disease [70] Potent anti-inflammatory agent in a model of multiple sclerosis (MS) [71] Cannabigerol derivative VCE-003.2 protects against mutant huntingtin-induced neurodegeneration [72] Cannabigerol derivative VCE-003 can be used in the treatment of human immune diseases [73] Potent against MRSA strains (MIC—2 µg/mL) * [54] Plays a neuroprotective role in the treatment of Huntington’s disease (HD) [74]
Cannabichromene	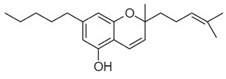	CB1—0.71 ^g^ CB2—0.256 ^g^ [45]	Potent against MRSA strains (MIC—8 µg/mL) * [54] Inhibit the proliferation of a hyper-proliferating human keratinocyte cell line in the treatment of psoriasis [55] Anti-inflammatory properties [75]

MIC-Minimum Inhibitory Concentration; * Antibacterial Activity against MRSA USA300; *K_i_*-inhibitory constant. ^a^ Assay—[3H]CP55-940 binding assay; Cell type-CHO cell membrane/Human. ^b^ Assay—[3H]CP55-940 binding assay; Cell type-CHO cell membrane/Mouse. ^c^ Assay—[3H]CP55-940 binding assay; Cell type-CHO cell membrane/Rat. ^d^ Assay—[3H]CP55-940 binding assay; Cell type-Whole-brain membranes/Mouse. ^e^ Assay—[3H]CP55-940 binding assay; Cell type-Cortical brain membranes/Rat. ^f^ Assay—[3H]CP55-940 binding assay; Cell type-CHO cell/Human. ^g^ Assay—[3H]CP55-940 binding assay; Cell type-Sf9 cells/Human.

**Table 2:** List of schedule 1 synthetic cannabinoids with their structures and adverse effects.

Name	Structure	Receptors	Adverse Effects
CP47,497 (5-(1,1-dimethylheptyl)-2-[(1R,3S)-3-hydroxycyclohexyl]-phenol)	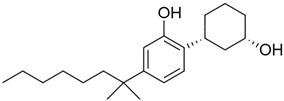	CB1 and CB2 agonist [149]	Increased heart rate, high/low blood pressure, coughing, and, vomiting [150]
JWH-018 (1-pentyl-3-(1-naphthoyl)indole)	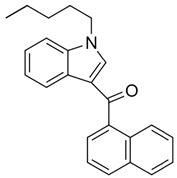	CB1 and CB2 agonist [149]	Psychosis [151], hallucinations, vertigo, paresthesias, shaking, shivering, hypertension, dry mouth, vomiting, mydriasis, hypokalemia, extrasystoles, tachycardia, conjunctival hyperemia [134], and ischemic stroke [135]
JWH-073 (1-butyl-3-(1-naphthoyl)indole)	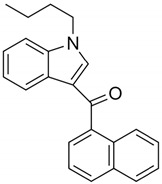	CB1 and CB2 agonist [149,152]	Altered mood and perception, red or bloodshot eyes, nausea, vomiting, listlessness, fever, sweating, and dryness of the mouth [150]
JWH-019 (1-hexyl-3-(1-naphthoyl)indole)	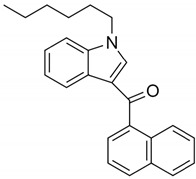	CB1 and CB2 agonist [152]	Confused speech, unstable appearance [150]
JWH-250 (1-pentyl-3-(2-methoxyphenylacetyl)indole)	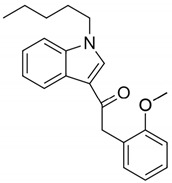	CB1 and CB2 agonist [149]	Dilated unresponsive pupils and bloodshot eyes [153]
JWH-081 (1-pentyl-3-[1-(4-methoxynaphthoyl)]indole)	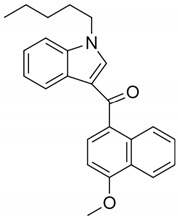	CB1 and CB2 agonist [152]	Slurred speech [153]
JWH-122 (1-pentyl-3-(4-methyl-1-naphthoyl)indole)	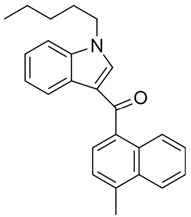	Agonist at CB1 and CB2 [154]	Hallucinations, disorientation, sedation, anxiety, agitation, tachycardia, hypertension, dyspnea, nausea, vomiting, hyperglycemia, and hypokalemia [155]
AM2201 (1-(5-fluoropentyl)-3-(1-naphthoyl)indole)	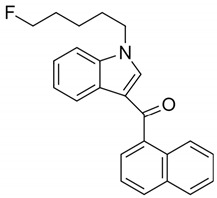	Agonist at CB1 and CB2 [156]	Convulsions [157], Excitatory behavior, xerostomia, chest pain, severe dyspnea, tachycardia (150 beats/min), and mild hypertension [158]
AM694 (1-(5-fluoropentyl)-3-(2-iodobenzoyl)indole)	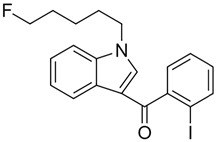	Agonist at CB1 [159]	Agitation, hallucination, anxiety, and paranoia [159]
JWH-203 (1-pentyl-3-(2-chlorophenylacetyl)indole)	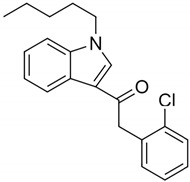	Agonist at CB1 and CB2 [156]	Head twitching, slurred speech, hallucination, dilated unresponsive pupils and bloodshot eyes [153]

**Table 3:** Two synthetic CBs MDMB-4en-PINACA and 4F-MDMB-BICA concerning EU. Reprinted from ref [160].

	MDMB-4en-PINACA	4F-MDMB-BICA
Structure	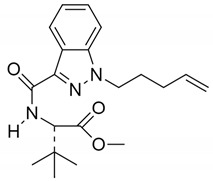	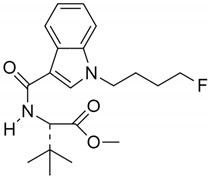
Molecular Formula	C_20_H_27_N_3_O_3_	C_20_H_27_FN_2_O_3_
Form	Yellow powder	White powder [163]
Availability	Since 2017	Since 2020
Cases reported	768 seizures as of October 2020. 11 acute non-fatal poisonings in the United Kingdom (UK) 4 confirmed deaths were reported in total by Sweden and UK	108 seizures as of October 2020 21 deaths reported by Hungary between May and August 2020

## Data Availability

Data is sharing not applicable.
